# Post-polymerization photografting on methacrylate-based monoliths for separation of intact proteins and protein digests with comprehensive two-dimensional liquid chromatography hyphenated with high-resolution mass spectrometry

**DOI:** 10.1007/s00216-015-8615-4

**Published:** 2015-03-24

**Authors:** Rudy J. Vonk, Sam Wouters, Andrei Barcaru, Gabriel Vivó-Truyols, Sebastiaan Eeltink, Leo J. de Koning, Peter J. Schoenmakers

**Affiliations:** 1 Analytical-Chemistry Group, van’t Hoff Institute for Molecular Sciences, University of Amsterdam, Science Park 904, 1098 XH Amsterdam, The Netherlands; 2 Department of Chemical Engineering, Vrije Universiteit Brussel, Pleinlaan 2, 1050 Brussels, Belgium; 3 Mass Spectrometry of Biomacromolecules, Swammerdam Institute for Life Sciences, University of Amsterdam, Science Park 904, 1098 XH Amsterdam, The Netherlands

**Keywords:** Photografting, Organic monoliths, Experimental design, Two-dimensional separation, Ion-exchange chromatography

## Abstract

Post-polymerization photografting is a versatile tool to alter the surface chemistry of organic-based monoliths so as to obtain desired stationary phase properties. In this study, 2-acrylamido-2-methyl-1-propanesulfonic acid was grafted to a hydrophobic poly(butyl methacrylate-*co*-ethylene glycol dimethacrylate) monolith to create a strong cation exchange stationary phase. Both single-step and two-step photografting were addressed, and the effects of grafting conditions were assessed. An experimental design has been applied in an attempt to optimize three of the key parameters of the two-step photografting chemistry, i.e. the grafting time of the initiator, the monomer concentration and the monomer irradiation time. The photografted columns were implemented in a comprehensive two-dimensional column liquid chromatography (^*t*^LC × ^*t*^LC) workflow and applied for the separation of intact proteins and peptides. A baseline separation of 11 intact proteins was obtained within 20 min by implementing a gradient across a limited RP composition window in the second dimension. ^*t*^LC × ^*t*^LC with UV detection was used for the separation of cytochrome *c* digest, bovine serum insulin digest and a digest of a complex protein mixture. A semi-quantitative estimation of the occupation of separation space, the orthogonality, of the ^*t*^LC × ^*t*^LC system yielded 75 %. The ^*t*^LC × ^*t*^LC setup was hyphenated to a high-resolution Fourier transform ion cyclotron resonance mass spectrometer instrument to identify the bovine serum insulin tryptic peptides and to demonstrate the compatibility with MS analysis.

**Electronic supplementary material:**

The online version of this article (doi:10.1007/s00216-015-8615-4) contains supplementary material, which is available to authorized users.

## Introduction

With the progressing complexity of the samples to be analysed, the quest for chromatographic systems with high separation power is increasingly relevant. One of the main criteria to assess whether complex samples can be analysed is the maximum number of peaks that can be separated in a single run, i.e. the peak capacity. To obtain higher peak capacities, comprehensive two-dimensional (2D) separation methods, such as time-based (^*t*^LC × ^*t*^LC, using a combination of columns) and spatial (^*x*^LC × ^*x*^LC, using a flat separation bed) have been developed. ^*x*^LC × ^*x*^LC has often been studied at low pressures in the form of thin-layer liquid chromatography (TLC) [1, 2]. The fundamental advantage of spatial separations in terms of analyses times is that the second-dimension separations are all performed in parallel. In contrast, in the online column-based ^*t*^LC × ^*t*^LC approach, all second-dimension separations are performed sequentially. Each fraction needs to be separated on the second-dimension column before the next fraction is injected. As a result, the first-dimension separation is usually very slow. In both ^*t*^LC × ^*t*^LC and ^*x*^LC × ^*x*^LC approaches, the first- and second-dimension separation systems should be completely orthogonal to make full use of the available peak capacity [3]. In time-based separations, the column dimensions can be optimized [4] and the stationary phases can be independently chosen. LC × LC, using a combination of SCX and RP as retention mechanisms and hyphenated to a mass spectrometer, has been demonstrated to be useful in identification of peptides [5, 6].

In our research, we are working on the development of an ^*x*^LC × ^*x*^LC device. The wide variety of organic-based monoliths that have been developed in the past decade may be attractive when stationary phases have to be created with very different (“orthogonal”) retention mechanisms. Different stationary phase chemistries can be created (locally) by post-polymerization reactions on the surface of the monolith, which makes organic-based monolithic stationary phases potentially suitable for use in both dimensions in LC × LC. Post-polymerization modification of the monolithic stationary phase can be performed by radical-induced grafting which can be initiated in many different ways, including chemical, photochemical, plasma-induced techniques and enzymatic grafting [7]. Photochemically induced post-polymerization surface modification was already demonstrated in the 1950s by Oster and Shibata [8]. More recently, it has been applied to LC stationary phases [9]. Two-step sequential photografting was shown to have advantages compared with single-step (direct) photografting. The two-step process precludes the formation of homopolymers which can cause blocking of the pores [10] and resulted in higher grafting yields [11].

**Fig. 1:**
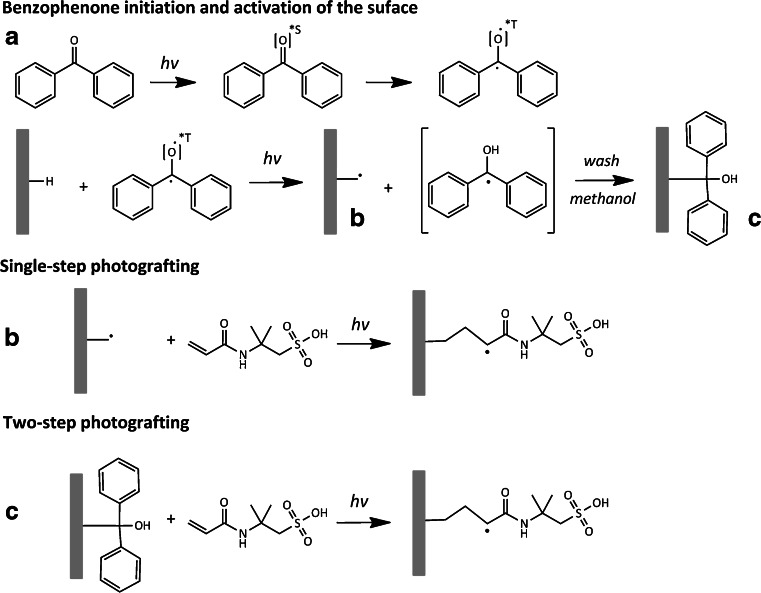
Proposed post-polymerization photografting mechanism to a poly(BMA-*co*-EDMA) monolithic surface. **A** The activation of the initiator followed by abstraction of hydrogen from surface. **B** For the single-step approach, the AMPS is grafted directly on the monolithic surface. **C** For the two-step sequential approach, the semi-pinacol radical is attached to the surface in the first step to be replaced by the grafted AMPS in the second step. Side reactions that could occur are two semi-pinacol radicals can combine to form dimers (termination) and surface radicals can be terminated by semi-pinacol radicals without attachment of function monomers (see also [10, 11, 13])

Ranby et al. [12, 13] discussed the mechanism of the photografting of several functional monomers by using benzophenone (BP) as initiator to absorb hydrogen atoms from the substrate, forming a radically active surface. BP molecules in the ground state absorb photons in the far UV region. When BP is transformed from the excited singlet state (S_1_) to triplet state (T_1_) via intersystem crossing, protons are abstracted from the polymer monolithic entity leaving substrate radicals (Fig. **1**a) that can subsequently be reactive with either monomers carrying the desired functional moiety leading to grafted chains (single-step) [14] (Fig. **1**a, b) or the semi-pinacol radical can be added to the surface (two-step). For the two step, after irradiating the columns with the initiator in the next step, the BP is replaced by the monomer [11], as shown in Fig. **1**a, c. This approach could be worthwhile to explore for locally adapting monolithic stationary phases in a ^*x*^LC × ^*x*^LC spatial device aimed at creating orthogonal separation mechanisms starting from the same base monolith. Since the engineering of an ^*x*^LC × ^*x*^LC chip is challenging, we explored the post-polymerization process in a time-based setup to later apply in the spatial device. Surface modification via post-polymerization has been rigorously optimized for the grafting of membranes, but not for the application in liquid chromatography. Although this photografting approach has previously been used for creating “orthogonal” stationary phases [11, 15, 16], to the best of our knowledge, no systematic study has yet been performed to establish optimal conditions for post-polymerization photografting of monolithic columns applied in LC.

Krenkova et al. [17] observed undesirable hydrophobic interactions when they applied a two-step photografting method of 2-acrylamido-2-methyl-1-propanesulfonic acid (AMPS) to the hydrophobic poly(glycidyl methacrylate-*co*-ethylene glycol dimethacrylate) (GMA-*co*-EDMA) monolith. To reduce the hydrophobic character of the resulting surface, they applied a multi-step photografting approach by first grafting a hydrophilic monomer (poly(ethylene glycol) methacrylate) and, subsequently, grafting a functional AMPS monomer to the stationary phase. With this approach, they showed an increased IEX-binding capacity. However, by increasing the number of post-polymerization steps, unwanted blockage can occur more easily. To obtain the highest possible binding capacity with fewer photografting steps, we investigated the post-polymerization grafting via two grafting approaches, i.e. a single-step approach that includes monomer and initiator in the grafting solution and a two-step approach that allows the covalent attachment of the initiator, followed by a graft polymerization reaction from the pore surface. Optimization of the grafting conditions of the two-step approach was systematically investigated using a central composite design (CCD) [18]. This experimental design was used for optimization of two-step post-polymerization photografting of AMPS to a stationary phase containing butyl methacrylate copolymerized with ethylene glycol dimethacrylate (poly(BMA-*co*-EDMA)). For the photografting reaction, the initiator concentration [11, 19], monomer concentration [11, 12], grafting time [20], energy dose [19], solvent [11, 21, 22] and temperature [12, 22] have been reported to influence the grafting yield of diverse monomers to various membrane substrates. For the current study, the number of positions at which BP can be attached in the first step is limited by the surface area of the polymerized monolith. Therefore, the concentration and the irradiation time are parameters which can influence the amount of BP attached to the surface. However, at high concentrations, BP absorbs much of the UV light. Ma et al. have found that a concentration of BP of 5 % was a concentration at which the BP was active and no significant absorbance of UV light was observed, which was why in the current study the BP concentration was kept constant at 5 % (*w*/*w*) [11]. The irradiation time was varied to find the optimal grafting conditions for the initiator. In the second grafting step, the concentration of the monomer and the grafting time were found to have an influence on the grafting yield on membranes [12]. Furthermore, high-density networks of grafted monomer, which were shown in previous studies to result in large surface areas, are unattractive since our final goal is to analyse proteins, which cannot enter the fine maze of grafted chains [23]. Increased grafting temperatures are known to increase the grafting yield [12, 22], but we were not able to perform grafting reactions at elevated temperatures. After considering all these previous studies, we fixed the solvent composition (to keep the monolith swollen) and the temperature. Rather than optimizing individual parameters, we investigated the grafting time of the initiator (*t*_1_), the monomer concentration (*c*_*m*_) and the monomer irradiation time (*t*_2_) as variables in the optimization of the photografting simultaneously using an experimental design.

The grafted columns obtained from this optimization study were used in a comprehensive ^*t*^LC × ^*t*^LC setup to show the potential of a near-orthogonal combination of a grafted cation exchange column and an (ungrafted) poly(styrene-*co*-divinylbenzene) (S-*co*-DVB) RPLC column. The “nano-scale” photografted columns (internal diameter 100 μm) were used in the first dimension (^1^D) in combination with a “capillary scale” (internal diameter 0.8 mm) S-*co*-DVB column in the second dimension (^2^D). For analyses of tryptic-digested cytochrome *c*, tryptic-digested bovine serum albumin (BSA) and tryptic-digested mixture of six proteins (PMD), the ion-exchange and reversed phase conditions were taken from literature [24, 25] and adapted for online coupling of the two methods using a UV detector. Finally, the applicability of the SCX × RPLC setup was demonstrated with the analyses of a BSA digest by hyphenation to a high-resolution Fourier transform ion cyclotron resonance mass spectrometer (FTICR-MS).

## Experimental

### Chemicals and materials

Butyl methacrylate (BMA, 99 %), ethyleneglycol dimethacrylate (EDMA, 98 %), styrene (S, >99.5 %), divinylbenzene (DVB, 80 %), 2-acrylamido-2-methyl-1-propanesulfonic acid (AMPS, 99 %), 2,2′-azobisisobutyronitrile (AIBN, 98 %), 1-decanol (99 %), cyclohexanol (99 %), toluene (99.9 %), 3-(trimethoxysilyl)propyl methacrylate (γ-MPS, 98 %), 2,2-dimethoxy-2-phenylacetophenone (DMPA, 99 %), benzophenone (BP, >99 %), *tert*-butanol (*t*-butanol, >99.5 %), aluminium oxide, benzyltrimethylammonium chloride (BTMAC, 97 %), propylparaben, bradykinin_1-5_ (>97 %), neurotensin (>90 %), angiotensin II human (>93 %), bovine insulin B-chain oxidize (>80 %), ribonuclease A from bovine pancreas, myoglobin from equine heart (>90 %), lysozyme from chicken egg white (>90 %), carbonic anhydrase from bovine erythrocytes, cytochrome *c* from equine heart (>95 %), human transferrin (>98 %), β-lactoglobulin B from bovine milk, β-lactoglobulin A from bovine milk (>90 %), catalase from bovine liver, myoglobin from equine heart (>90 %), α-lactalbumin from bovine milk (>85 %) and conalbumin from chicken egg were purchased from Sigma-Aldrich (Zwijndrecht, The Netherlands). ACTH_18-39_ was purchased from Bachem (Bubendorf, Switzerland). Trypsin (Gold) was purchased from Promega (Madison, USA). Methanol (MeOH), acetonitrile (ACN), acetone and tetrahydrofuran (THF, >99.8 %) were purchased from Biosolve (Valkenswaard, The Netherlands). Sodium hydroxide (NaOH) was purchased from Merck (Darmstadt, Germany). Hydrochloric acid (HCl) (37 % *v*/*v*) was obtained from Acros (Geel, Belgium). Milli-*Q* water (18.2 MΩcm) was produced by a Sartorius Arium 611UV Ultrapure Water System (Goettingen, Germany). BMA, EDMA, S and DVB were purified by passing the liquid precursors through a bed of activated basic alumina. Tryptic digest of the protein mix (PMD, 100 pmol, lyophilized) originating from six proteins (viz*.* cytochrome *c,* lysozyme, alcohol dehydrogenase, bovine serum albumin, apo-transferrin, β-galactosidase) was purchased from Thermo Scientific (Breda, The Netherlands).

UV-transparent fused silica capillary (TSU, 0.1 mm internal diameter) used to prepare the BMA-*co*-EDMA-*gr*-AMPS columns was purchased from Polymicro Technologies (Phoenix, AZ, USA). For the preparation of S-*co*-DVB columns, glass-lined tubes (0.8 mm internal diameter × 50 mm length) were purchased from Da Vinci Europe (Rotterdam, The Netherlands).

### Sample preparation

A tryptic digest of cytochrome *c* was prepared in house by dissolving the protein in 0.1 M Tris–HCl at pH 7.5 prior to digestion with trypsin (1:20 *w*/*w*) for 16 h at 37 °C. The bovine serum albumin (BSA) digest was prepared by dissolving the protein in 0.1 M Tris–HCl at pH 7.5 with 10 % ACN. The alkylation of sulfhydryl groups was performed by additions of dithiothreitol for 30 min at 55 °C followed by the addition of iodoacetamide for 20 min at room temperature. Next, the BSA was digested with trypsin (1:50 *w*/*w*) for 16 h at 37 °C. The digests were desalted using OMIX-C18 reversed phase pipette tips (Agilent, Amstelveen, The Netherlands) eluted with 0.1 % TFA in 50 % ACN (*v*/*v*) in water and dried in a vacuum centrifuge. Prior to analyses, the samples were dissolved in 0.05 % TFA in water.

### Monolithic columns: preparation and photografting

Prior to polymerization, the internal surface of the fused silica capillary was functionalized to allow the monolith to be covalently bonded to the wall [26]. For functionalization, the fused capillary was flushed for consecutive periods of 30 min at a flow rate of 2 μL min^−1^ with 1 M NaOH solution, water, 0.2 M HCl solution and, again, water. Thereafter, the columns were dried using a flow of nitrogen. The capillary was next flushed with a 10 % (*v*/*v*) γ-MPS in toluene for 1 h followed by removal of unreacted chemicals by flushing with acetone. The glass-lined tubing was treated with identical steps, except that a flow rate of 10 μL min^−1^ was applied. Finally, the capillaries and glass-lined tubing were dried by purging with nitrogen.

The methacrylate-based BMA-*co*-EDMA monolith was synthesized in situ in pre-treated fused silica capillaries using cyclohexanol and 1-decanol as porogenic solvents. Typically, a 200-mm-long capillary was filled with the polymerization mixture, and UV polymerization was performed with irradiation at 365 nm for 5 min (batch I) or 254 nm for 20 min (batch II) (Spectroline, Distrilab, Leusden, The Netherlands). After polymerization, the monolithic columns were thoroughly flushed with MeOH before analyses to remove all unreacted monomers and porogenic solvents. The preparation of the S-*co*-DVB monoliths in glass-lined tubing was described in detail in our previous work [24].

The post-polymerization functionalization of the BMA-*co*-EDMA monoliths was performed using a single-step and two-step approach as described previously in literature [10]. For the single-step approach, the columns were flushed for 30 min with 2 % BP (*w*/*w*) as initiator and 4 % AMPS (w/w) dissolved in MeOH to water (2:1) and irradiated at 365 nm for 10 and 20 min, respectively. For the two-step process, the columns were flushed for 30 min with 5 % BP (*w*/*w*) as initiator dissolved in MeOH followed by UV irradiation for variable times (first variable, *t*_1_). After the initiator was attached to the surface, the columns were flushed with MeOH for at least 20 column volumes. Next, the various concentrations of AMPS (second variable, *c*_*m*_) were dissolved in water before the addition of *t*-butanol. The ratio of water to *t*-butanol was kept constant (25:75 % *w*/*w*) to dissolve both the functional monomer and the photoinitiator [9] and to maintain the monolith in the swollen state [7, 22]. The columns were flushed with the AMPS solution for 30 min. Next, UV irradiation was performed during various times (third variable, *t*_2_). Both photografting steps in the two-step approach were performed using UV light at 254 nm. After UV grafting, the BMA-*co*-EDMA-*gr*-AMPS columns were thoroughly flushed with MeOH for at least 40 column volumes to flush out any unreacted chemicals. All solutions were sonicated and purged with nitrogen for 10 min prior to use to ensure homogeneity and to remove all oxygen.

### Study of photografting with experimental design

We applied a central composite experimental design to optimize the grafting conditions so as to obtain the highest possible grafting yield when applying the two-step photografting (specified below). The number of experiments for a full factorial experimental design is 2^*n*^ with *n* the number of parameters (variables) considered. For each of the three grafting parameters, reasonable practical ranges were chosen guided by the conditions used in previous photografting studies. For the grafting time of the initiator (*t*_1_), optimization was tested between 4 and 10 min, with a central point of 7 min and the corresponding axial points [18]. A similar strategy was followed to select different monomer grafting times (*t*_2_) and monomer concentrations (*c*_*m*_). In pilot experiments, higher *c*_*m*_ (above 15 %) resulted in the blockage of the monomers. A total of 15 different grafting conditions were established as shown in the Supplementary material section S1. Grafting experiments were performed at least in duplicate at each condition. The repeatability (*n* = 11) was investigated by repeated experiments at *t*_*1*_ = 4 min, *t*_*2*_ = 7 min, *c*_*m*_ = 11.3 %, which were the conditions of the experimental design resulting in the highest binding capacity for benzyltrimethylammonium chloride (BTMAC) (see “Functionalization of the pore surface via photografting”). Because grafting reactions only affect the surface of the monolith, direct evidence for changes in the chemistry is difficult to obtain. We attempted to confirm the presence of sulphate groups by FTIR analyses of grafted samples, but we were not able to observe sulphate bend vibrations at 1150 cm^−1^.

### Instrumentation and chromatographic conditions

Analyses were performed on an Ultimate 3000 RSLCnano system (Thermo Scientific, Breda, The Netherlands) and on an Agilent HP 1100 cap-LC (Agilent Technologies, Amstelveen, The Netherlands). In the cap-LC setup, two UV detectors (model 200, Linear Instruments, Fremont, CA, USA) were used in series to accurately determine the delivered flow rate. The binding capacity of the resin was obtained through breakthrough analyses with a 50-μL sample loop to inject BTMAC solution dissolved in mobile phase A consisting 40 % (*v*/*v*) ACN. The columns were flushed with 50 mM NaCl dissolved in mobile phase A (20 column volumes) before each new breakthrough experiment. A Shimadzu CBM20 was used as AD-convertor, and data were recorded using Shimadzu VP client 7.4.

Analyses of intact proteins, protein digests and peptides were performed on the Ultimate 3000. The system consisted of a dual LC-pump, a thermostatted autosampler, an air-heated column oven compartment with two integrated 10-port switching valves, one valve equipped with fraction collection loops (either 1, 3.3 or 5 μL, depending on the sample fraction volume from the ^1^D) and the other was used for online desalting. The UV detector was equipped with either a 3-nL Z-shaped detector cell for the 1D capillary measurements (typically operated at 3–5 μL min^−1^) or a 180-nL Z-shaped detector cell for the 2D measurements (typical second-dimension flow rate (^2^ *F*) of 50–200 μL min^−1^). For measuring the ion-exchange capacity with the intact proteins, the mobile phase A1 consisted of 10 mM potassium acetate buffer pH 5.0 with 25 % (*v*/*v*) ACN to suppress any possible hydrophobic interactions [27]. Mobile phase B1 was 500 mM KCl dissolved in A1. Separations were performed using a 5-min gradient from 2.5 to 500 mM KCl. For the online 2D setup with UV detection for analysing proteins, 5-μL fraction collection loops were installed and the ^1^D flow rate (^1^ *F*) was set to 0.5 μL min^−1^. For the separation of protein digests, the mobile phase A1′ was 10 mM potassium phosphate at pH 2.7 containing 25 % ACN, while mobile phase B1′ was 0.25 M KCl dissolved in A1′ [25]. In LC × LC-UV analyses for both the intact proteins and protein digests, the RP mobile phase A2 consisted of 0.05 % (*v*/*v*) TFA in H_2_O, while mobile phase B2 consisted of 20:80 % (*v*/*v*) H_2_O/ACN with 0.04 % (*v*/*v*) TFA using 1-μL fraction collection loops. Prior to use, the eluents were degassed by sonication for 20 min. Gradient separations were performed at 60 °C using full-loop injection. Depending on the nature of the samples, the gradient program (time and starting/final compositions) was changed so as to obtain effective gradient windows. Data were recorded using Chromeleon 6.80 (SR11) chromatography management system software.

### LC × LC-FTICR MS/MS analysis of protein digest

LC × LC-MS/MS data were acquired with a Bruker ApexUltra FTICR-MS/MS (Bruker Daltonics, Bremen, Germany) equipped with a 7-T magnet and a nano-electrospray Apollo II DualSource coupled to the Ultimate 3000 RSLCnano system. Trypsin-digested BSA sample was injected in a slightly adapted chromatographic system. The ^2^D gradient was adapted by reducing the ^2^D flow rate and increasing the modulation time to 12 min. To collect the larger fractions, two fraction collection loops of 3.3 μL (0.13 mm × 250 mm) were installed. To enhance the compatibility with the electrospray ionization, the 0.05 % TFA was replaced by 0.1 % (*v*/*v*) formic acid (FA). This is a weaker ion-pairing agent for the RP analyses (resulting in less retention), but yielded a higher electrospray ionization efficiency. A second 10-port valve was used to remove the salt plugs from the ^1^D gradient before electrospray ionization. The salt from the ^1^D fractions was flushed to waste with mobile phase A1 for 1 min prior to the start of the gradient while the peptides were trapped on top of the analytical column. Post-column flow splitting resulted in a nano-electrospray flow rate of 500 nL min^−1^ (see Supplementary material section S2 for a detailed overview of the valve configuration). An absolute amount of ca. 1 μg of the BSA tryptic peptide mixture was injected.

MS spectra were recorded each 0.5 s for accurately monitoring the elution profiles of the peptides. For identification of peptides, MS/MS data were recorded with a duty cycle of less than 2 s so that a maximum of three data-dependent Q-selected peptide ions were fragmented in the hexapole collision cell at an argon pressure of 6 × 10^−6^ mbar (at the ion gauge). Both MS precursor peptide ions and the corresponding MS/MS fragment ions were detected in the ICR cell at a resolution of up to 60,000. Instrument mass calibration was better than 1 ppm over the *m*/*z* range of 250 to 1500. The raw FTICR-MS/MS data were processed with the MASCOT DISTILLER program, version 2.4.3.1 (64 bits), MDRO 2.4.3.0 (MATRIX science, London, UK), including the Search toolbox and the Quantification toolbox. Peak-picking parameters for both MS and MS/MS spectra were optimized for a mass resolution of up to 60,000. Peaks were fitted to a simulated isotope distribution with a correlation threshold of 0.7, with minimum signal to noise of 2. The processed MS/MS data were searched with MASCOT server program 2.3.02 (MATRIX science, London, UK) against the Swissprot protein database (release 2012-09) [28], taxonomy Mammalia. Trypsin/P was used as enzyme and one missed cleavage was allowed. Oxidation of methionine was allowed as a variable modification. The peptide tolerance was set to 10 ppm, and the peptide fragment mass tolerance was set to 0.03 Da. MASCOT peptide identification was set to a cutoff of 20. The identified BSA tryptic peptides are listed in Table S1 in the Electronic Supplementary Material (ESM).

Raw LC × LC-FTICR-MS data were processed with the Data Analyser 4.1 software of Bruker. All mass spectra (total 7300) were extracted from the chromatogram, and for each spectrum, the monoisotopic masses of the peptides were determined using Bruker’s peak recognition technology SNAP II. For the array of 7300 spectra, these masses were exported in Mascot generic file (mgf) format with the corresponding retention time and ion abundances summed over all isotopes of all detected ion charge states. The identification of the peptides was realized by matching the mass and LC retention with those of the peptides identified with the corresponding LC-FTICRMS/MS data. The mgf MS data array enabled construction of the elution profiles of all identified peptides with a digital resolution of about 0.5 s. MATLAB^™^ version 2012b was used to create the 3D plots shown in Fig. 5 from the mgf data array. The peak maxima of peptides were automatically detected based on monoisotopic peptide masses (±40 ppm) using an in-house MATLAB script.

## Results and discussion

### Column-to-column repeatability prior to photografting

**Fig. 2:**
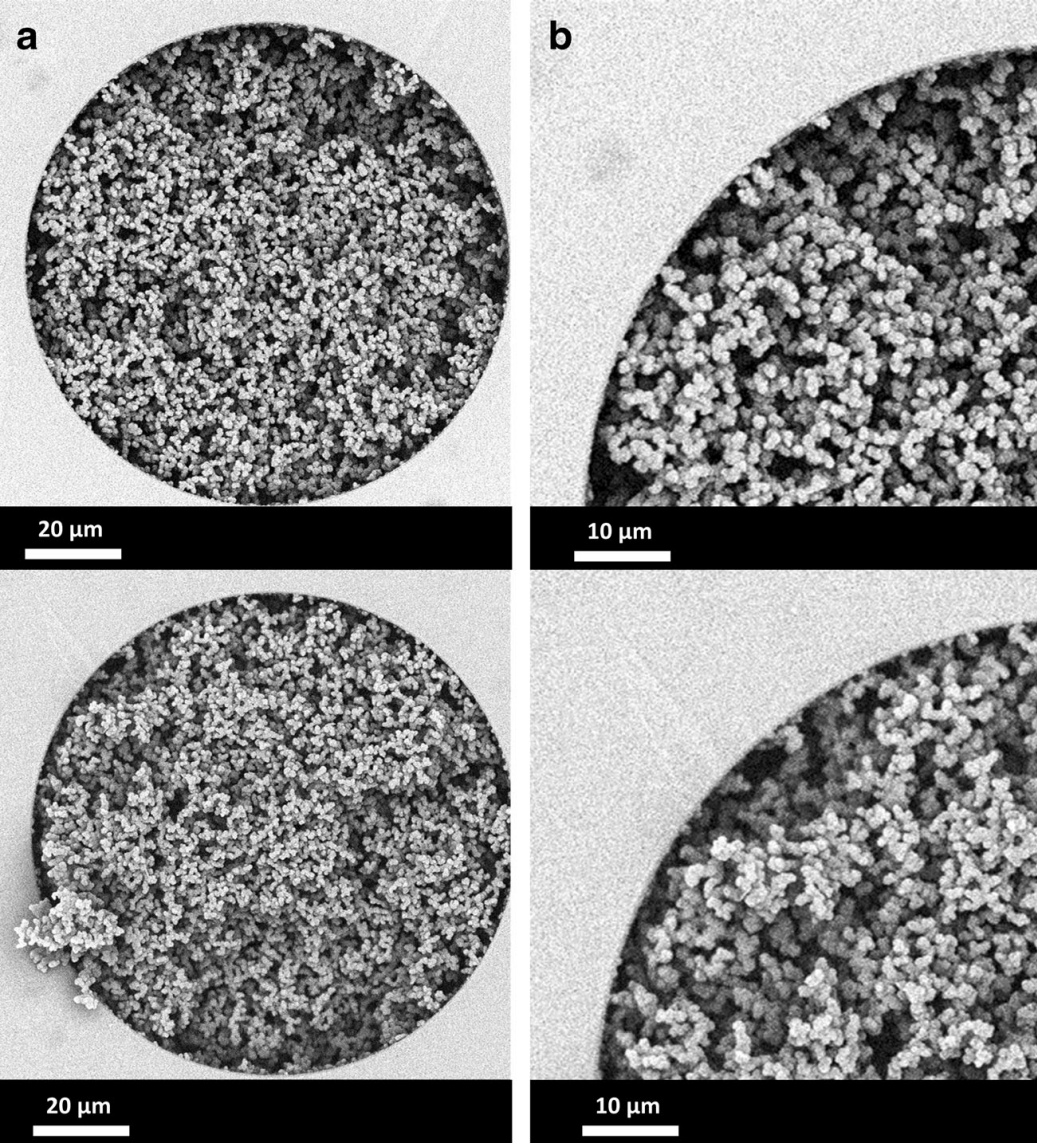
Scanning electron micrographs **A** of the cross-section of fused silica capillaries and **B** region to the wall and the bulk globular region. On the *top* and *bottom*, different monolithic columns both contain porous poly(butyl methacrylate-co-ethylene dimethacrylate) monoliths from the first batch

Before the post-polymerization photografting was applied to adapt the surface chemistry, the macroporous structure of the monolithic columns was tuned via optimization of the porogen ratio (cyclohexanol/1-decanol). In Fig. 2, the scanning electron micrograph images are shown of the cross-section (Fig. 2a) of two randomly selected columns of the first batch of monolithic columns (top and bottom). The monoliths feature typical homogenously distributed interconnected globules well attached to the wall (Fig. 2b). No significant variation in globule size and macropore size could be observed between the various monolithic columns.

The capillary monolithic columns were characterized prior to the post-polymerization functionalization by measuring the pressure drop across the column length that can be converted to the normalized permeability (*K*_*v*)_ of each column. Table 1 provides the permeability of the columns prepared in two batches. Batch I was prepared using a monomer-to-porogen ratio of 33:67 %. By using the Grubb’s outlier test (significance level of *α* = 0.01), only one outlier was detected and, hence, discarded. The variation in *K*_*v*_ (213 (±24) × 10^−15^ m^2^) corresponds to a RSD = 11.4 %, which is comparable with the data presented in the repeatability RSD of 10.8 % obtained by Geiser et al. [29]*.* To enhance the surface area (create smaller globules), the polymer monolithic capillary columns in batch II were polymerized using a monomer-to-porogen ratio of 40:60 %, resulting in columns with a slightly lower permeability *K*_*v*_ = 127 (±15) × 10^−15^ m^2^, corresponding to a RSD = 11.7 %, which is also comparable to the first batch and the previous described batch in literature [29]. More details concerning the monolithic columns can be found in the ESM section S1.

**Table 1:** Column-to-column repeatability in terms of permeability data determined for two batches of monolithic capillary columns prior to pore surface functionalization via photografting; batch II was polymerized on several days

	Batch I	Batch II		
#	*K*_*v*, day 1_ (×10^−15^ m^2^)	*K*_*v*, day 1_ (×10^−15^ m^2^)	*K*_*v*, day 2_ (×10^−15^ m^2^)	*K*_*v*, day 3_ (×10^−15^ m^2^)
1	222	121	132	113
2	259	101	112	134
3	197	119	125	144
4	181	115	145	131
5	203	125	114	140
6	179	109	119	147
7	189	117	117	142
8	236	107		140
9	216	136		125
10	209	134		128
11	218	101		169
12	200	121		137
13	205	101		149
14	216	119		142
15	260	115		146
16				142
17				144
18				146
AVG ± STDEV	209 ± 24	117 ± 12	124 ± 12	139 ± 15
RSD %	11	10	9	9

### Functionalization of the pore surface via photografting

**Fig. 3:**
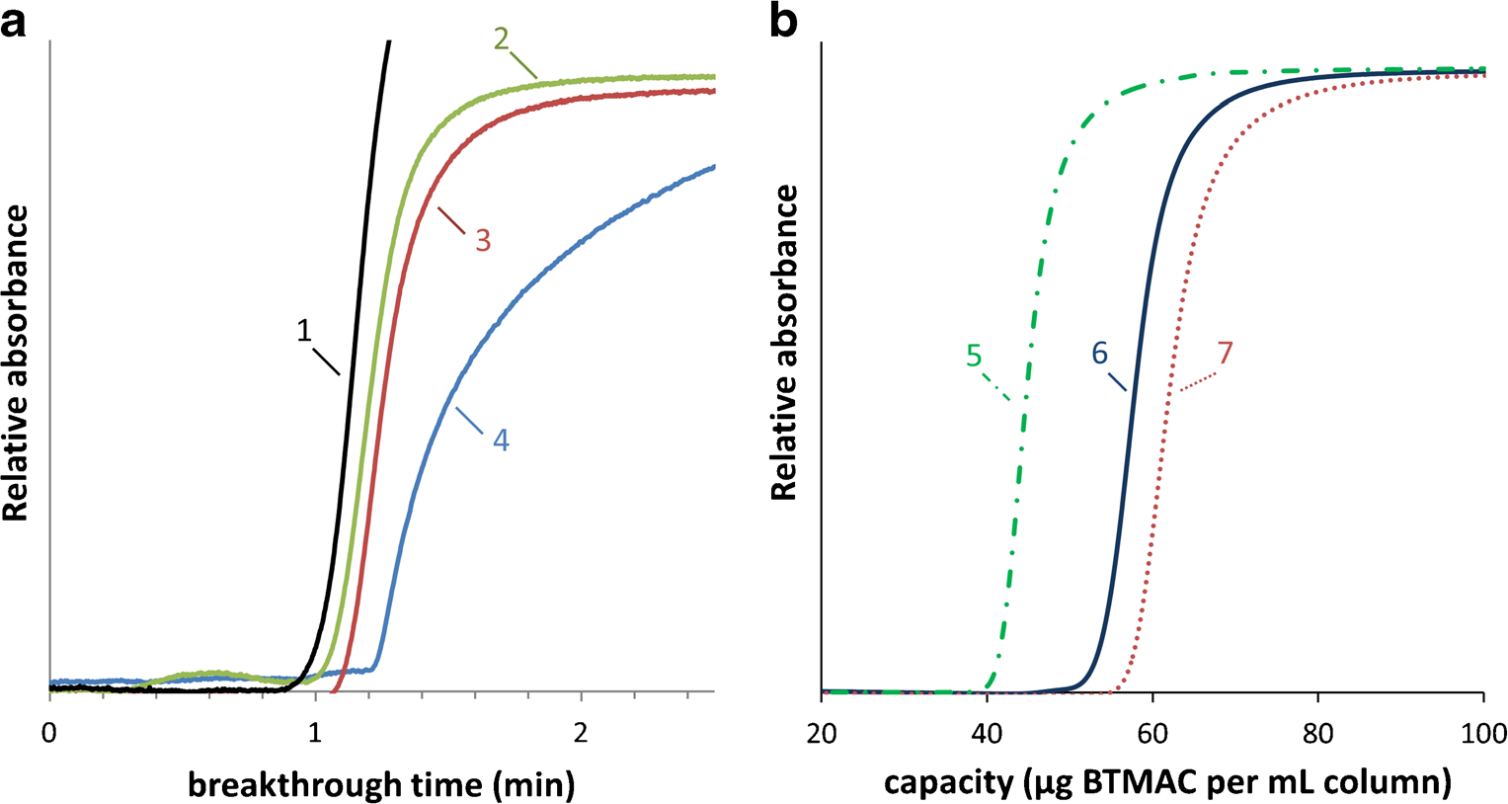
Determination of grafting yield through the breakthrough of BTMAC. In **A**, difference in grafting yield between single-step and two-step approaches in relation to uracil (unretained marker), normalized to 100 mm column length and in **B** breakthrough curves at varying monomer grafting times (*t*_2_). *1* uracil, *2* single-step 10 min, *3* single-step 20 min, *4*–*7* two-step with *4 t*_1_ = 4 min, *t*_2_ = 7 min and *c*_*m*_ = 11.3 %, *5 t*_1_ = 4 min, *t*_2_ = 2 min, *c*_*m*_ = 7.5 %, *6 t*_1_ = 4 min, *t*_2_ = 7 min, *c*_*m*_ = 7.5 % and *7 t*_1_ = 4 min, *t*_2_ = 12 min, *c*_*m*_ = 7.5 %

Two photografting strategies, i.e. a single-step approach and a sequential two-step photografting process (see Fig. **1** for reaction mechanisms), were assessed using BP as photoinitiator to functionalize the pore surface of the methacrylate polymer globules. The single-step photografting was first explored using UV light at 365 nm. Although the absorbance of UV is higher at lower UV wavelength and thus result in more efficient grafting, longer grafting times using 365 nm should lead to post-polymerization functionalization of the pore surface [14]. Grafting efficiency was visualized by the analyses of the breakthrough of BTMAC. Figure 3a shows the breakthrough curves of uracil and BTMAC. On a generic monolith (ungrafted), the BTMAC breakthrough was identical to the uracil breakthrough suggesting that the addition of 25 % ACN is sufficient to suppress hydrophobic interactions (data not shown). Increased single-step photografting times (20 min) resulted in increased grafting efficiency (Fig. 3a). Nevertheless, a low efficiency was obtained which could be caused by low initiation rate of BP at 365 nm as well as by the relative low surface area of the monolith (batch I). To enhance the ion-exchange capacity, a generic monolith with a higher surface area was developed (batch II). However, the single-step grafting at 254 nm (5 min irradiation) resulted in blockage of the pores due to the formation of homopolymers. As an alternative, a two-step photografting approach was investigated in more detail. In Fig. 3a, the difference in grafting yield, between the single-step (10 and 20 min at 365 nm) and two-step (*t*_1_ = 4 min, *t*_2_ = 7 min and *c*_*m*_ = 11.3 % at 254 nm), is shown. To optimize the grafting conditions of the two-step process, an experimental design was applied. Grafting efficiency was quantified by the analyses of the breakthrough of BTMAC (50 % height). Although the permeability was reduced at all of the 15 sets of grafting conditions constituting the experimental design, no conditions resulted in blockage of the columns so that all grafting conditions could be used for modelling of the post-polymerization grafting reaction. The quantitative breakthrough of BTMAC was used as the response for the modelling. In Fig. 3b, a sub-set of the breakthrough curves is shown with varying grafting times of the monomer (*t*_2_).

When the grafting was repeated (*n* = 11; see ESM Table S2), eight columns were successfully grafted; the other three columns were lost due to mishaps during flushing of the columns in the sequential photografting steps. Based on the breakthrough experiments and the peak capacity (*n*_*c*_) measurements obtained for the separation of ovalbumin, carbonic anhydrase and lysozyme, the variation in the resulting columns was found to be reasonably small, with RSD values of 6 and 15 % for the binding capacity and the *n*_*c*_, respectively. The precision of the breakthrough experiments was modest (15 % RSD). This effect, in combination with the limited degrees of freedom of the experimental design, prevented us from accurately predicting optimal two-step photografting conditions.

### Online comprehensive two-dimensional analyses

The combination of a BMA-*co*-EDMA-*gr*-AMPS column with a S-*co*-DVB column was used in online comprehensive SCX × RPLC for the analyses of a number of model proteins and for the analyses of protein digests. The total peak capacity (^2*D*^ *n*_*c*_) of the setup can be calculated by multiplying the peak capacity of the first dimension (^1^ *n*_*c*_) (the number of fractions collected) with the peak capacity of the second dimension (^2^ *n*_*c*_) [30]. The latter can be calculated by Eq. 1 [31]

1 $$ {}^2{n}_c=\frac{{}^2{t}_G}{{}^2W}+1 $$

Using the second-dimension gradient time (^2^ *t*_*G*_) and the average peak width at baseline (^2^ *W*).

For the protein digest samples, the first-dimension gradient was adapted for the peaks to cover as much of the separation window as possible. For each 3 min, a loop was filled with first-dimension effluent and subsequently emptied into the second-dimension column. To prevent loss of analytes during the online analyses, the first-dimension flow rate did not exceed the 0.2 μL min^−1^.

**Fig. 4:**
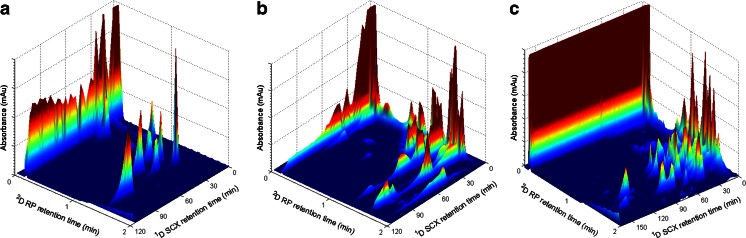
LC×LC-UV analyses of protein digest samples using a linear ^1^D gradient from 1.25 to 247.5 mM KCl in 80 min and ^2^ *φ* from 0.008 to 0.40 in 2 min for **A** cytochrome *c* digest, **B** BSA digest and **C** for a PMD with ^1^D gradient of 120 min

To make maximum use of the peak capacity available with the system, we aimed to collect 2 to 3 fractions from each first-dimension peak [30]. Therefore, the ^1^D gradient was run from 1.25 to 247.5 mM KCl in 80 min for the cytochrome *c* digest. The ^2^D gradient ran from 1 to 50 % B2 (*φ*_ACN_ from 0.08 to 0.40) in 2 min, allowing 1 min for equilibration of the column between injections. As can be seen in Fig. 4a, the relative simple digest of cytochrome *c* resulted in 12 peaks throughout the LC × LC chromatogram. Because elution of the peptides was not observed in some of the ^1^D fractions, the effective sample peak capacity (peak capacity in the region of the chromatogram was the peaks that can be observed; ^2*D*^ *n*_*c,* sample_) was estimated as 27 × 39 = 1053. This is only true if the ^1^D band width is smaller than the fraction collection time [30]. On the same setup, the more complex BSA digest and digested protein mixture (PMD) were also analysed. The separations are shown in Fig. 4b, c. For the PMD sample, the ^1^D gradient was extended to 120 min. The contour plots reveal the absence of highly charged hydrophilic peptides in the two samples. Many analytes elute non-retained from the ^1^D column. The PMD analyses resulted in a ^2*D*^ *n*_*c,s*ample_ of 1800 (46 × 39), which is about 40 % less than the value obtained by Wagner et al. [32] using similar retention mechanisms and total analyses time, but a more complex setup. They used two RP columns in parallel and four (instead of two) collection loops. This allowed longer ^2^D gradient times, resulting in higher 2D and overall peak capacities. The presented setup requires less sophisticated instruments, but still provides high peak capacities in online mode within an analyses time of 2.5 h. To demonstrate the applicability in proteomic approaches, the LC × LC setup was hyphenated to an FTICR-MS/MS instrument. This necessitated some modifications to the ^2^D gradient, both in terms of mobile phase composition and gradient time. The strong ion-pairing agent TFA, which is attractive from a perspective of chromatographic performance, was replaced in mobile phases A2 and B2 by FA (0.1 % *v*/*v*), which is more attractive from a perspective of MS ionization efficiency. The ^2^D flow rates were reduced, and gradient times were concomitantly increased to allow sufficient time for MS/MS cycles. With a ^1^D gradient from 1.25 mM to 247.5 mM KCl of 40 min in combination with a ^2^D gradient from 1 to 40 % B2 in 3.5 min, a sequence recovery of 38 % was obtained for BSA. To recover more peptides, the ^1^D gradient was adapted to include 5 min isocratic at 0.5 mM KCl followed by linear increase to 125 mM in 12 min and an additional linear increase to 249.5 mM KCl in 60 min. The ^2^D gradient (^2^ *F* = 50 μL min^−1^) started with 1 min isocratic at ^2^ *φ*_start_ = 0.008 for each run, followed by a 9-min gradient to ^2^ *φ*_end_ = 0.4, allowing equilibration for 2 min (at an increased flow rate, ^2^ *F*_eq_ = 170 μL min^−1^) before each following run. The “salt plugs” (first-dimension mobile phase), which eluted unretained from the ^2^D column, were flushed to waste. The valve setup (shown in the ESM, Fig. S2) was tested with a set of five peptides with a wide range of hydrophobicity indices [33] (e.g. bradykinin_1-5_, neurotensin, angiotensin II, ACTH_18-39_ and bovine insulin B-chain) to cover the ^2^D elution window as efficiently as possible. In Table 2, the concentration of modifier at elution (^2^ *φ*_e_) is shown for each compound. Additionally, in this table, a comparison is made for the elution strength (^2^ *φ*_e_) at the moment of elution for the peptides under the gradient elution using FA and TFA as ion-pairing agent used in LC × LC-UV experiments.

**Table 2:** Comparison of mobile phase composition (*φ*_e_) at moment of elution for peptides on the RP column with different ion-pairing agents. In the LC × LC-UV experiments, 0.05 % TFA was added to mobile phase A2, while for the LC × LC-MS/MS experiments 0.1 % FA was added to mobile phase A2

Peptide	Hydrophobicity index^a^	^2^ *φ*_e*,TFA*_	^2^ *φ*_e*,FA*_
Bradykinin_1-5_	7.5	0.05	0.02
Angiotensin	16.8	0.29	0.22
Neurotensin	27.2	0.38	0.31
ACTH_18-39_	47.9	0.47	0.43
Bovine insulin	78.7	0.56	0.53

^a^Calculation of the hydrophobicity index was based on [32]

**Fig. 5:**
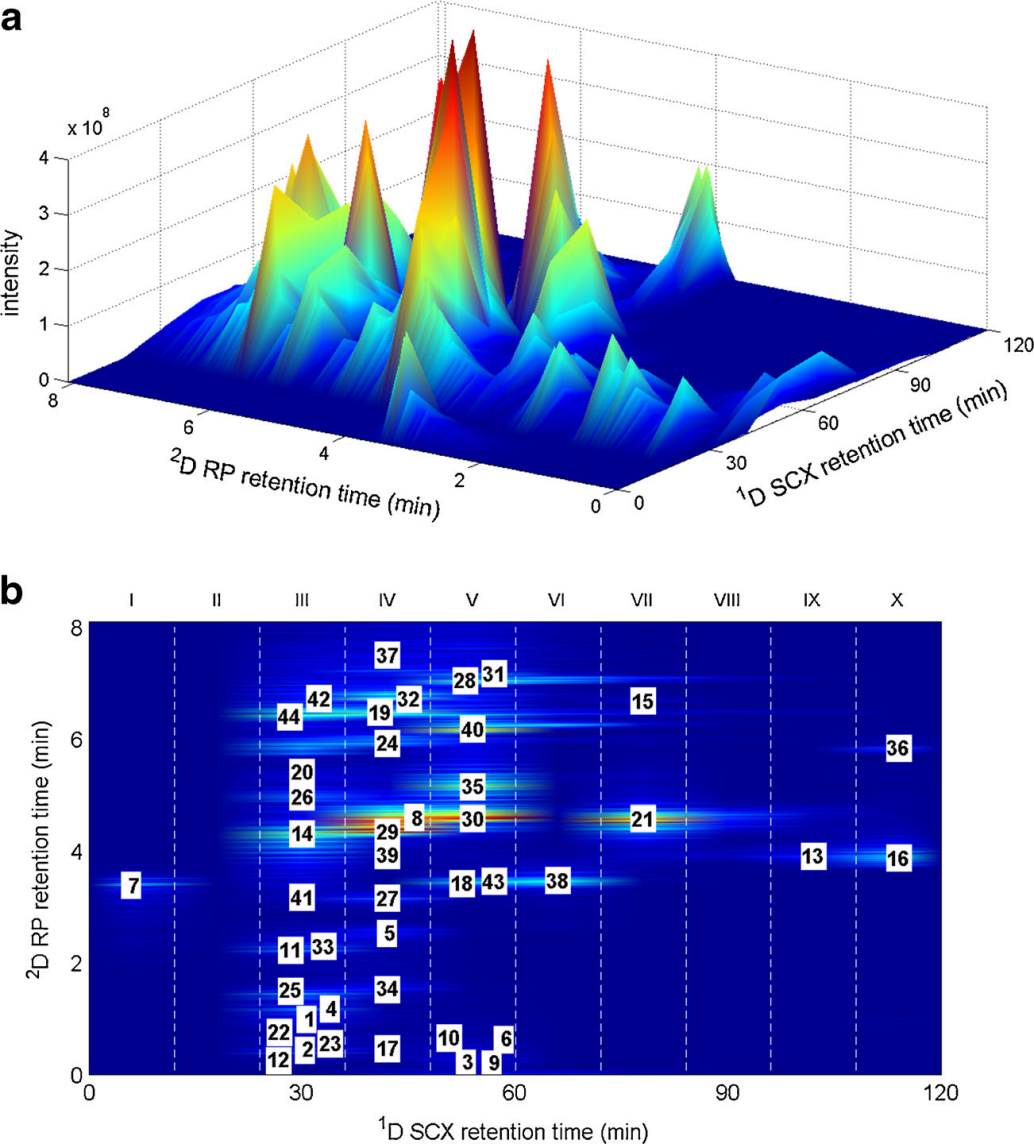
**A** LC×LC-FTICR-MS TIC of identified peptides of BSA digest. **B** Top view with each identified peptide labelled at its peak maximum. The *roman numbers* indicate the SCX fractions collected. The chromatograms were obtained using a ^1^D gradient starting with 5 min isocratic elution at 0.5 mM KCl followed by a linear increase to 125 mM in 12 min and an additional linear increase to 249.5 mM in 60 min at a flow rate of 150 nL min^−1^ and ^2^ *φ* (ACN) from 0.008 to 0.40 in 9.0 min, with 3 min desalting and equilibration of the RP column. Further information regarding the peptides can be found in the ESM Table S1

The trapping efficiency at the top of the analytical column is limited for hydrophilic peptides. Therefore, peptides with a hydrophobicity lower than bradykinin_1-5_ (e.g. ATEEQLK) will not be observed in the present LC × LC-MS setup. The modification of the ^2^D gradient resulted in increased peak widths at baseline (^2^ *W*) from 3 s in the LC × LC-UV experiments to 20 s for the LC × LC-MS/MS experiments and the second-dimension *n*_*c*_ decreased from 39 to 27. The LC × LC-MS/MS setup resulted in the identification of 96 % of the BSA peptides in a total of ten IEX fractions with a ^2*D*^ *n*_*c,s*ample_ of 270. The total sequence coverage of the BSA digest sample was found to be 60 %. This illustrates the potential of the current approach for the structure elucidation of complex proteomic samples. However, improvements in the LC × LC-MS setup, such as miniaturizing the ^2^D flow rate to ensure direct hyphenation need to be addressed. The most hydrophilic peptides were probably lost due to inadequate trapping during the online desalting step on the ^2^D column. A detailed overview of the identified peptides is provided in the ESM Table S3. A total ion current (TIC) chromatogram of the identified BSA peptides is shown in Fig. 5a. In Fig. 5b, a top view of the 2D chromatogram is shown displaying the identified peptides (sorted based on their monoisotopic masses) at their maximum intensity at the appropriate position in the separation space. In the present experiments, most of the peptides were contained in fractions 3 to 5 from the ^1^D SCX separation. In the first SCX fraction, only the C-terminus peptide (LVVSTQTALA) with a 1^+^ charge was observed. The “best peak” chromatogram of the BSA digest sample can be found in the ESM Fig. S3. More ions were observed, but these were either not selected for MS/MS fragmentation or were not identified as known peptides.

In the LC × LC-UV experiments, the ^2^D flow rate was increased to reduce the extra-column band broadening in the ^2^D separation. This ^2^D flow rate was too high to allow direct coupling with the ESI-nanoprobe. Despite reducing the flow rate fourfold, the high flow rate is still a drawback of the current setup because only 1 % of the injected sample was sent to the ESI-nanoprobe after post-column splitting. Reducing the dimensions of the ^2^D column to “nano-format” (i.e. 75 μm i.d.) will reduce consumption of precious samples. This can be achieved via modulation (focussing) between the first and second dimensions, allowing minimization of the ^2^D column dimension and resulting in an online LC × LC-FTICR-MS/MS setup for proteomics with an increased (mass) sensitivity.

Recently, Camenzuli and Schoenmakers described a new method to quantify the orthogonality of an LC × LC experiment [34]. The application of this method to the chromatogram shown in Fig. 4b yields an orthogonality of 75 % (for more details, see ESM section S3). Due to the absence of highly charged, polar peptides in this BSA digest sample, the orthogonality did not reach 100 %. Nevertheless, the value indicates a good coverage of the separation space.

**Fig. 6:**
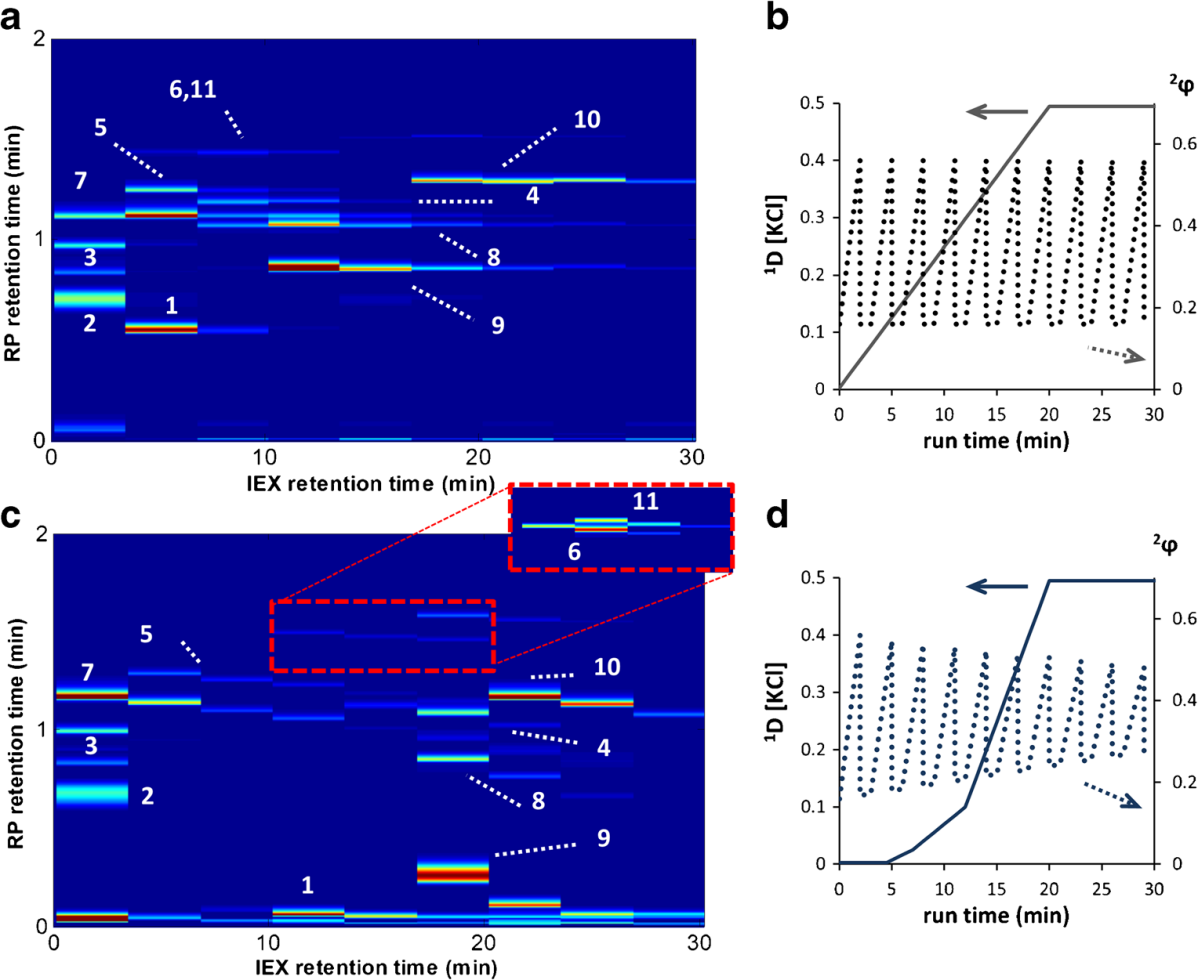
**A** Separation of 11 proteins by LC×LC-UV using the method as shown on the right. **B** Gradient conditions for separation shown in **A** on the left axis the concentration of counter ion and on the right axis ^2^ *φ*. **C** Separation of the same protein mixture as in **A** using the optimized method **D** with zoom-in illustrating the baseline separation of proteins 6 and 11. **D** Gradient conditions for separation shown in **C**. Proteins eluted were *1* ribonuclease A, *2* ovalbumin, *3* carbonic anhydrase, *4* transferrin, *5* α-lactalbumin, *6* β-lactoglobulin B, *7* trypsinogen, *8* lysozyme, *9* cytochrome *c*, *10* myoglobin, *11* β-lactoglobulin A

The separation of intact proteins is shown in Fig. 6a. No separation was possible of β-lactoglobin A (protein #11) and β-lactoglobin B (protein #6) with the standard method applying a linear ^1^D gradient and an identical ^2^D gradient for each fraction, i.e. an identical starting concentration ACN (^2^ *φ*_start_) and gradient windows (^2^ *∆φ*). Furthermore, with the chosen model proteins, no elution was observed in the ^1^D fractions collected at a low ^2^ *φ*. Bedani et al. showed that an increased coverage of the separation space could be obtained with the implementation of a “narrowed” gradient in the second dimension [35]. Two critical proteins β-lactoglobin A and β-lactoglobin B were separated after implementing the shifting ^2^D gradient and a modified ^1^D gradient. The ^1^D gradient started isocratically at 1.25 mM KCl and was set to an exponential increase, and the ^2^D gradient was programmed with an increased ^2^ *φ*_start_ and decreasing ^2^ *φ*_end_ for each fraction, resulting in a decreased ^2^ *Δφ* for each fraction. The separation with the optimized method is shown in Fig. 6c, and the baseline separation of β-lactoglobin A and β-lactoglobin B is shown in the amplified region of this figure. The gradient programs of the non-optimized and optimized gradient are shown in Fig. 6b, d, respectively. The orthogonality can be accurately characterized for samples with more than 25 peaks [34], and therefore, we cannot quantify the orthogonality for the separation of 11 proteins. Nevertheless, the optimized method can be said to show a good coverage of the separation space and a good performance (^2*D*^ *n*_*c,*sample_ = 500, within 30 min analyses time).

## Concluding remarks

The photografted BMA-*co*-EDMA-*gr*-AMPS columns, using the two-step approach, could be used for IEX separations of proteins before grafting (BMA-*co*-EMDA columns) RP separation was possible. Despite significant efforts, the post-polymerization grafting conditions could not be rigorously optimized because the experimental design did not yield a sufficiently precise model for the effect of the post-polymerization grafting conditions. The variation in the individual points could be reduced to values less than <7 % (RSD), suggesting that post-polymerization grafting may be a robust method for adapting the surface chemistry of monolithic (BMA-*co*-EMDA) stationary phases. The grafted columns were implemented in the first dimension of an online comprehensive LC × LC-UV setup with RP monolithic columns in the second dimension. This system was successfully applied for the separation of cytochrome *c* digest, BSA digest and a digest of a protein mixture, yielding a total sample peak capacity exceeding 1000 within 90 min reaching 1800 for a 2.5-h analysis time. The LC × LC system was coupled to an FTICR-MS/MS instrument, demonstrating the ability to obtain qualitative information on a BSA digest. LC × LC-MS/MS runs were complete within 2 h, and a sequence coverage of 60 % for the BSA peptides was obtained. Modulation between the first- and second-dimension, which may be achieved by focussing and desalting on trap columns before analyses on the ^2^D column [36], may allow reducing the ^2^D column diameter and, thus, the second-dimension flow rate. Low ^2^D flow rates allow the best hyphenation with the FTICR-MS/MS detector because all the injected samples can be introduced into the MS. Additionally, online desalting will allow analysing relatively hydrophilic peptides, which are flushed to waste with the salt plugs in the current setup. An improved LC × LC-MS/MS setup is attractive for online analyses of samples that are currently analysed in offline MudPit approaches [25], dramatically reducing the sample handling steps. Progress in the direction of more complex samples can be envisaged by implementing modulation and by reducing the gap between the (high) optimum flow rate for the second dimension in LC × LC and the (low) optimum flow rate of the nano-electrospray MS interface.

Apart from the IEX separations of peptides, the grafted columns may also be used for the separation of intact proteins. The coverage of the separation space was increased for the separation of 11 intact proteins within 30 min, while two critical proteins (β-lactoglobin A and β-lactoglobin B) could be separated by implementing an exponential first-dimension gradient and a narrowing second-dimension gradient (higher starting composition and smaller composition window for each subsequent run).

## Electronic supplementary material

Below is the link to the electronic supplementary material.

ESM 1 (PDF 458 kb)
