# Supplementary material for: Post-polymerization photografting on methacrylate-based monoliths for separation of intact proteins and protein digests with comprehensive two-dimensional liquid chromatography hyphenated with high-resolution mass spectrometry
Source: Anal Bioanal Chem. 2015 Mar 24;407(13):3817–29. doi: 10.1007/s00216-015-8615-4 (PMC4412387; doi:10.1007/s00216-015-8615-4)
Supplement: Supplementary file 1 — (PDF 458 kb) [file 216_2015_8615_MOESM1_ESM.pdf]

Analytical and Bioanalytical Chemistry

Electronic Supplementary Material

Post-polymerization photografting on methacrylate-based monoliths for separation of intact proteins and protein digests with comprehensive two-dimensional liquid chromatography hyphenated with high-resolution mass spectrometry

Rudy J. Vonk, Sam Wouters, Andrei Barcaru, Gabriel Vivó-Truyols, Sebastiaan Eeltink, Leo J. de Koning, Peter J. Schoenmakers

Aim

In this article we tried to optimize the two-step post-polymerization photografting conditions of 2-acrylamido-2-methyl-1-propanesulfonic acid (AMPS) to a monolithic butyl methacrylate copolymerized with ethylene glycol dimethacrylate (poly(BMA-*co*-EDMA)). Two different polymerization batches were investigated, details can be found in Table S1. Parameters to optimize the photografting conditions were selected based via an experimental design. Three grafting parameters, of the two-step post-polymerization photografting, were chosen to yield in the highest bindingcapacity of the resin (*i.e.* the grafting time of the initiator (t_1), the monomer concentration (c_m) and the monomer irradiation time (t_2)). All 15 different grafting conditions can be found in Table S2. The photgrafted columns were implemented in comprehensive two-dimensional liquid chromatography for the analyzes of proteins and protein digest. Finally we demonstrated the ability to hypenate the setup with a high-resolution Fourier-transform ion-cyclotron mass spectrometor (FTICR-MS/MS) for the analyses of a BSA digest.

Modeling of the photografting conditions using an experimental design

The quantitative breakthrough of BTMAC (50% height) was used as the response for the modelling. In Fig. S1A a sub-set of three grafting conditions (1-3, identical to conditions from Fig. 3B) were compared to the breakthrough capacity of the maximum predicted optimum condition (t_1 = 3.4 min, t_2 = 6.4 min, c_m = 8.1%) (5). This resulted in an experimental capacity which was higher than the predicted experimental capacity (4) (Y_{exp} = 100 µg per mL column volume *vs.* Y_{pred} = 65 ± 40 µg per mL column volume). We applied the quadratic model, without interaction, to calculate the predicted binding capacities at the various grafting conditions (Fig. S1C). The yellow color indicates high Y_{pred} and the grafting conditions resulting in the blue region result in a low Y_{pred}.

Table S1 Polymerization details (w/w %) of the two batches used for single-step photografting (batch I) and two-step grafting (batch II)

**Chemical**	**Batch I**	**Batch II**
BMA	13.3	16
EDMA	20	24
cyclohexanol	20.7	5
1-decanol	46	55
DMPA*	6	1
wavelength (nm)	365	254
Irradiation time (min)	5	20
n	15	61
K_v ($\times 10^{-15}$) m^2 (RSD)	213 ± 24 (11.4%)	127 ± 15 (11.7%)

* percentages of initiator with respect to the monomer concentration.

Table S2 Grafting conditions selected in the experimental design for optimization of post-polymerization grafting

Experiment	t_1 (min)	t_2 (min)	c_m (% (*w*/*w*))	c (mg/mL) *	*n*
1	2	4	5	67 ± 3	2
2	2	4	10	63 ± 11	2
3	2	10	5	57 ± 4	3
4	2	10	10	68 ± 1	2
5	6	4	5	56 ± 4	2
6	6	4	10	41 ± 3	2
7	6	10	5	51 ± 3	2
8	6	10	10	75 ± 11	2
9	4	7	7.5	62 ± 9	2
10	0.6	7	7.5	78 ± 2	3
11	7.4	7	7.5	59 ± 2	2
12	4	2	7.5	58 ± 5	2
13	4	12	7.5	47 ± 5	2
14	4	7	3.3	39 ± 9	2
15 **	4	7	11.3	89 ± 6	8

* Binding capacity of BTMAC in mg/mL (±STDEV), for modelling the grafting conditions

** These grafting conditions were used for addressing the repeatability of photografting conditions, the c_m according the experimental design should have been 11.7%

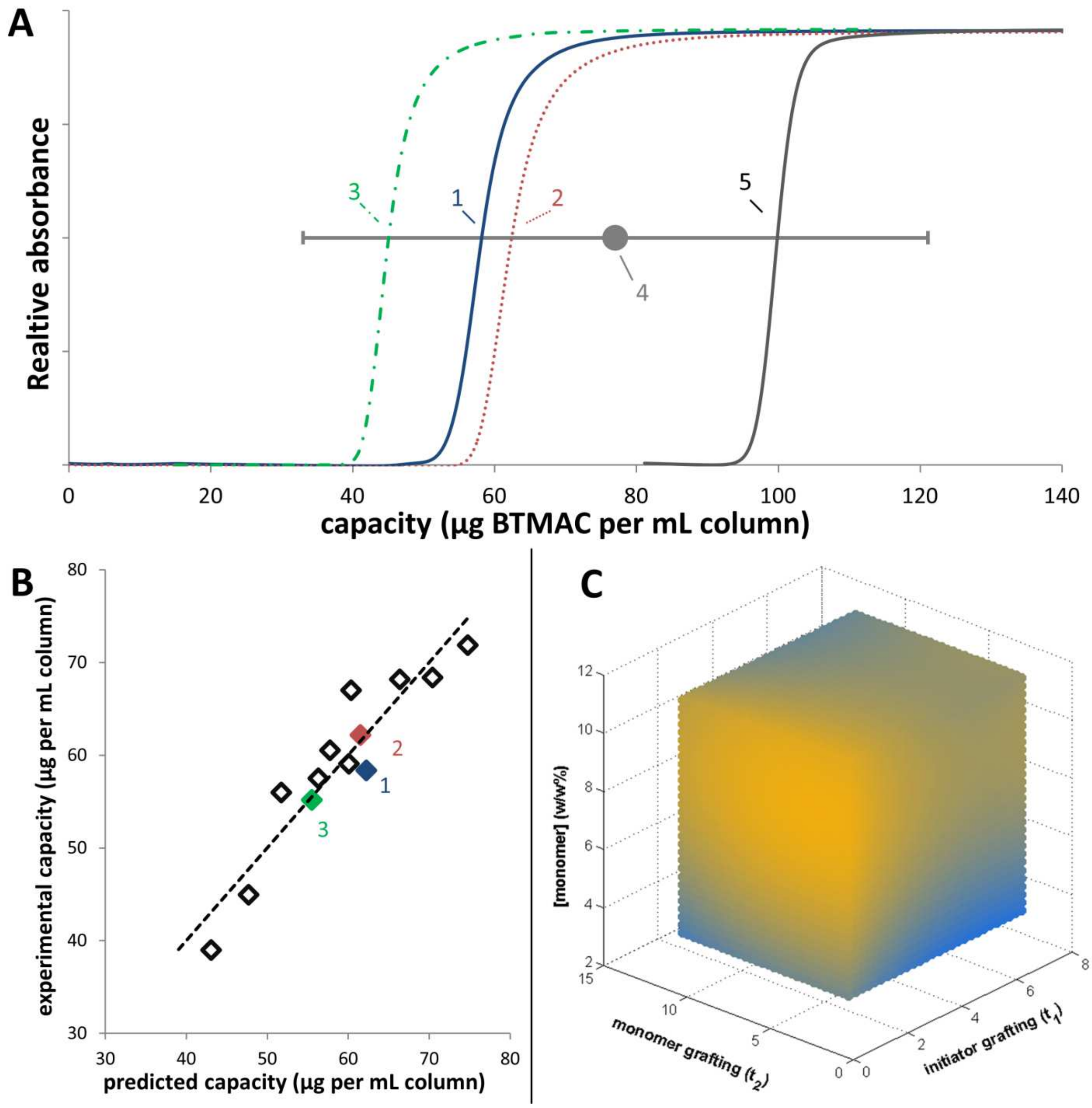

Fig. S1 Determination of grafting yield by the breakthrough of BTMAC. In A) Breakthrough curves at varied monomer grafting times (t_2). B) predicted *vs.* experimental binding capacity. C) Calculated grafting yield at all grafting conditions within the experimental design.

1) t_1 = 4 min, t_2 = 2 min, c_m = 7.5%, 2) t_1 = 4 min, t_2 = 7 min, c_m = 7.5% and 3) t_1 = 4 min, t_2 = 12 min, c_m = 7.5%, 4) theoretical breakthrough of optimal grafting point and 5) experimental breakthrough of optimal grafting point

Hyphenation of LC×LC with FTICR-MS/MS

In Fig. S2 the valve configuration is shown that was used for comprehensive two-dimensional LC combined with the FTICR-MS. The SCX-fractions were collected in loop I and II by switching the left valve accordingly. The collected fractions were injected in the ^{2}D effluent and online desalting was applied by trapping the peptides on the top of the analytical column, flushing the salt residue to waste. The ^{2}D effluent was split just before the MS such that the nano-electrospray flow did not exceed 500 nL·min^{-1}.

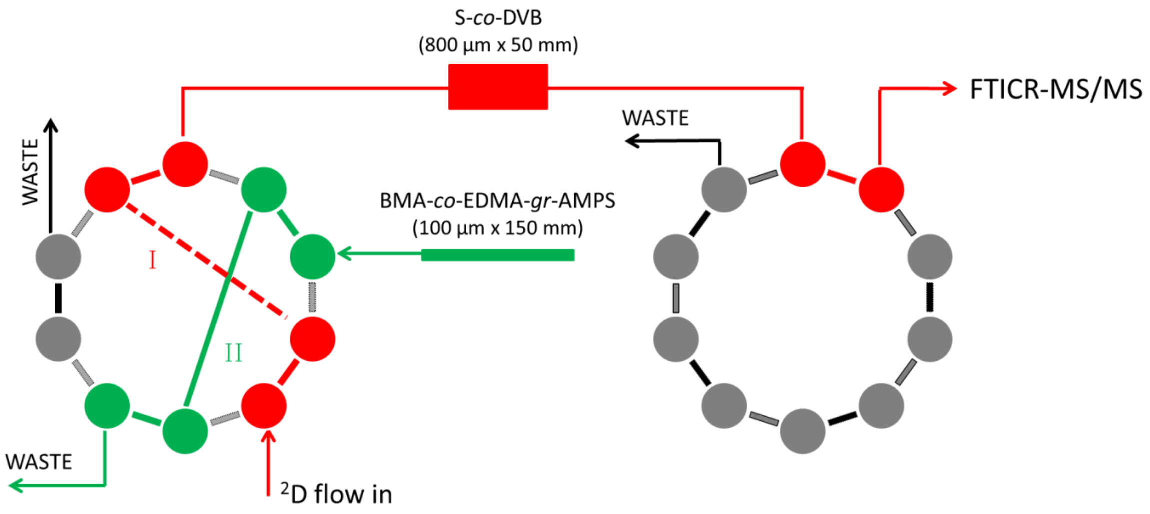

Fig. S2 Schematic overview of valves used in LC×LC-MS setup. The RP desalting was performed by switching valve B to the 10_1 position, elution was performed in the 1_2 position

Detailed information on the BSA-digest peptides identified by MASCOT is provided in Table S3. The peptides were sorted by their mono-isotopic mass. Details regarding their elution fraction of the ^{1}D (SCX) and ^{2}D (RP) elution time were provided. In Fig. S3 the best-peak chromatogram (BPC) and the total-ion current (TIC) of the BSA disgets were shown in comparision to the TIC of the identified peptides as was given in the main article in Fig. 4. The magenta arrows indicate extra observed peaks which might originated from non-identified peptides or from background matrix. Total sequence coverage of 60% was established.

Table S3 Overview of identified peptides from BSA digest analysed on the 2D LC×LC-MS/MS. Identification using MASCOT

Peptide	Peptide sequence	Calculated Mol. Mass (Da)	SCX fraction	RP ret time (min)
1	GACLLPK	757.4156	3	1.00
2	LVTDLTK	788.4644	3	0.42
3	LCVLHEK	897.4742	5	0.22
4	AEFVEVTK	921.4807	3	1.18
5	YLYEIAR	926.4861	4	2.53
6	DLGEEHFK	973.4505	5	0.32
7	LVVSTQTALA	1001.5757	1	3.38
8	QTALVELLK	1013.6121	4	4.58
9	NECFLSHK	1033.4651	5	0.22
10	SHCIAEVEK	1071.5019	5	0.30
11	EACFAVEGPK	1106.5066	3	2.22
12	CCTESLVNR	1137.4907	3	0.23
13	KQTALVELLK	1141.707	9	3.90
14	LVNELTEFAK	1162.6234	3	4.30
15	FKDLGEEHFK	1248.6139	7	6.65
16	HPEYAVSVLLR	1282.7034	10	3.87
17	ECCDKPLLEK	1290.5948	4	0.48
18	HLVDEPQNLIK	1304.7088	5	3.42
19	TVMENFVAFVDK	1398.6853	4	6.45
20	TVMENFVAFVDK + oxidation	1414.6803	3	5.38
21	SLHTLFGDELCK	1418.6864	7	4.57
22	YICDNQDTISSK	1442.6347	3	0.48
23	ETYGDMADCCEK	1477.516	3	0.67
24	LGEYGFQNALIVR	1478.7881	4	5.90
25	EYEATLEECCAK	1501.6065	3	1.45

Peptide	Peptide sequence	Calculated Mol. Mass (Da)	SCX fraction	RP ret time (min)
26	VPQVSTPTLVEVSR	1510.8355	3	4.95
27	DDPHACYSTVFDK	1553.6457	4	3.15
28	DAFLGSFLYEYSR	1566.7354	5	7.00
29	LKPDPNTLCDEFK	1575.7603	4	4.33
30	KVPQVSTPTLVEVSR	1638.9305	5	4.57
31	MPCTEDYLSLILNR	1723.8273	5	7.13
32	MPCTEDYLSLILNR + oxidation	1739.8222	4	6.68
33	YNGVFQECCQAEDK	1746.6978	3	2.30
34	ECCHGDLLECADDR	1748.6553	4	1.53
35	RPCFSALTPDETYVPK	1879.9138	5	5.13
36	HPYFYAPELLYYANK	1887.9195	10	5.82
37	VASLRETYGDMADCCEK	1889.785	4	7.51
38	NECFLSHKDDSPDLPK	1900.8625	6	3.47
39	NECFLSHKDDSPDLPK + Deaminated	1901.8465	4	3.92
40	LFTFHADICTLPDTEK	1906.9135	5	6.15
41	CCAADDKEACFAVEGPK	1926.791	3	3.17
42	DAIPENLPPLTADFAEDK	1954.992	3	6.50
43	ECCHGDLLECADDRADLAK	2246.9354	5	3.45
44	DAIPENLPPLTADFAEDKDVCK	2457.1733	3	6.40

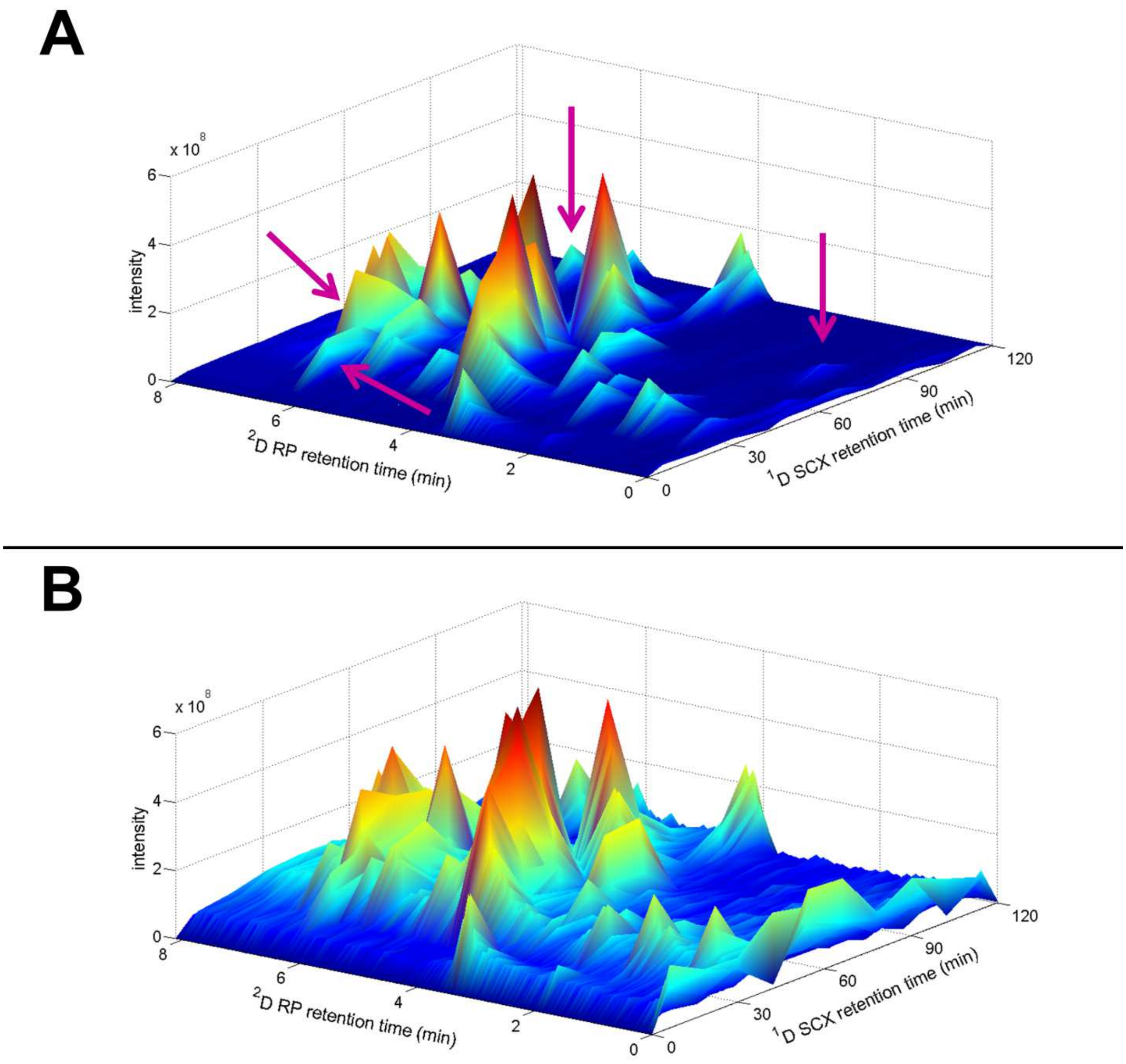

Fig. S3 BSA digest analyses on a LC×LC-FTICR-MS giving (A) BPC and (B) TIC. As comparison to Fig. 4 in which the TIC of identified peptides is shown. The chromatograms were obtained using a ^{1}D gradient starting with 5 min isocratic elution at 0.5 mM KCl followed by a linear increase to 125 mM in 12 min and an additional linear increase to 249.5 mM in 60 min at a flow rate of 150 nL/min and $^{2}\varphi$ (ACN) from 0.008 to 0.40 in 9.0 min, with 3 min de-salting and equilibration of the RP column

Orthogonality of obtained LC×LC chromatograms

The so called "asterisk" equation (A_0) considers all peaks in a 2D chromatogram by studying their positions with respect to four Z-lines (Z_-, Z_+, Z_1 and Z_2) [33]. The spreads around these lines (Z_n) are calculated for each component (i) by Eq. (1)-(4) and used to determine the orthogonality of the chromatogram

$$Z_- = \left|1 - 2.5\left|\sigma\{\,^1t_{R,norm(i)} - \,^2t_{R,norm(i)}\} - 0.4\right|\right| \quad (1)$$

$$Z_+ = \left|1 - 2.5\left|\sigma\{\,^2t_{R,norm(i)} - (1 - \,^1t_{R,norm(i)})\} - 0.4\right|\right| \quad (2)$$

$$Z_1 = 1 - \left|2.5 \times \sigma\{\,^1t_{R,norm(i)} - 0.5\} \times \sqrt{2 - 1}\right| \quad (3)$$

$$Z_2 = 1 - \left|2.5 \times \sigma\{\,^2t_{R,norm(i)} - 0.5\} \times \sqrt{2} - 1\right| \quad (4)$$

were the σ = standard deviation, for all peaks within the chromatogram and both the $^1t_{R,norm(i)}$ and $^2t_{R,norm(i)}$ are normalized for the 1st and 2nd dimension retention time for each (i) by Eq. (5)

$$t_{R,norm(i)} = \frac{t_{R(i)} - t_{R,first}}{t_{R,last} - t_{R,first}} \quad (5)$$

The percentage of the orthogonality for the chromatogram is calculated by Eq. 6

$$A_0 = \sqrt{Z_- \times Z_+ \times Z_1 \times Z_2} \quad (6)$$

The occupation-degree of the LC×LC separation-space, along with the number of peaks eluting in each zone, are parameters affecting the value of A_0. In a fully orthogonal separation, the spread of sample components around all lines will be maximized, resulting in A_0 = 100% [33]. In Fig. S4A a top view of the BSA digest separation obtained with LC×LC-UV experiments (Fig. 4B) is shown, together with the crossing Z-lines. In Fig S4B the 75 most abundant eluting peptides were selected and marked in the separation space. The peaks were chosen as uniquely as possible by only selecting peaks which did not occur in the previous ^{1}D fraction within accuracy specified precision (time window) of 0.04 min of the ^{2}D elution time. When a peak was observed in a ^{2}D

run the highest data point was selected at the specified retention time and eventual lower peaks, at the same retention time in the previous or following ^{2}D runs were discarded. For the example shown in Fig. S3B an orthogonality of 75% was obtained.

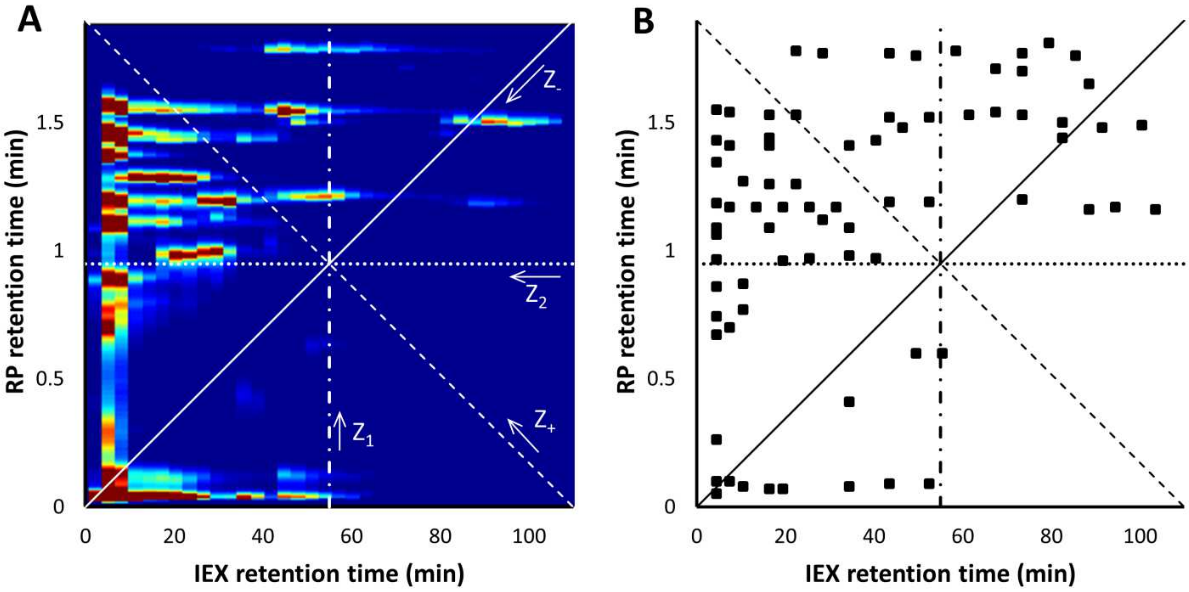

Fig. S4A) Top view of the comprehensive analyses of BSA digest, as show in Fig. 4B, with the crossing Z-lines B) overview of the 75 most abundant peptides (■) used for determination of the A^o-value
